# Micro-Expression-Based Emotion Recognition Using Waterfall Atrous Spatial Pyramid Pooling Networks

**DOI:** 10.3390/s22124634

**Published:** 2022-06-19

**Authors:** Marzuraikah Mohd Stofa, Mohd Asyraf Zulkifley, Muhammad Ammirrul Atiqi Mohd Zainuri

**Affiliations:** Department of Electrical, Electronic and Systems Engineering, Faculty of Engineering and Built Environment, Universiti Kebangsaan Malaysia, Bangi 43600, Selangor, Malaysia; p109858@siswa.ukm.edu.my (M.M.S.); ammirrulatiqi@ukm.edu.my (M.A.A.M.Z.)

**Keywords:** deep learning, convolutional neural networks, micro-expression analysis, emotion classification

## Abstract

Understanding a person’s attitude or sentiment from their facial expressions has long been a straightforward task for humans. Numerous methods and techniques have been used to classify and interpret human emotions that are commonly communicated through facial expressions, with either macro- or micro-expressions. However, performing this task using computer-based techniques or algorithms has been proven to be extremely difficult, whereby it is a time-consuming task to annotate it manually. Compared to macro-expressions, micro-expressions manifest the real emotional cues of a human, which they try to suppress and hide. Different methods and algorithms for recognizing emotions using micro-expressions are examined in this research, and the results are presented in a comparative approach. The proposed technique is based on a multi-scale deep learning approach that aims to extract facial cues of various subjects under various conditions. Then, two popular multi-scale approaches are explored, Spatial Pyramid Pooling (SPP) and Atrous Spatial Pyramid Pooling (ASPP), which are then optimized to suit the purpose of emotion recognition using micro-expression cues. There are four new architectures introduced in this paper based on multi-layer multi-scale convolutional networks using both direct and waterfall network flows. The experimental results show that the ASPP module with waterfall network flow, which we coined as WASPP-Net, outperforms the state-of-the-art benchmark techniques with an accuracy of 80.5%. For future work, a high-resolution approach to multi-scale approaches can be explored to further improve the recognition performance.

## 1. Introduction

A micro-expression is a human emotion expressed briefly, spontaneously, and unwillingly. Usually, there are emotions that people desire to keep hidden for a variety of reasons. Due to their subtlety and spontaneity, it is harder to conceal micro-expressions than to repress fake long-duration expressions. As a result, it is very difficult for a human to spot micro-expressions at a glance. However, slow-motion capturing of these micro-expressions using high-speed digital cameras allows us to play back the video for analysis purposes. In the early period of micro-expressions analysis systems development, handcrafted feature extraction techniques such as Histogram of Oriented Gradient (HOG) [1], Main Directional Mean Optical flow (MDMO) [2], Bi-Weighted Oriented Optical Flow (Bi-WOOF) [3], and Local Binary Pattern with Three Orthogonal Planes (LBP-TOP) [4] were used to extract the spatio-temporal information used for the automated recognition. In addition, all these conventional machine learning techniques are heavily dependent on designer experience in extracting the optimal set of features [5]. As the machine learning technology becomes more mature, researchers have turned to convolutional neural networks (CNNs) to extract the optimal set of features needed for micro-expression-based emotion classification.

**Figure 1:**
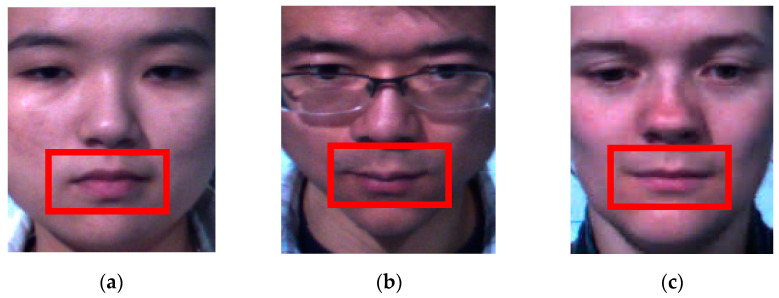
Differences in facial muscle movement for happy emotion among the test subjects: (**a**) subject 1; (**b**) subject 2; (**c**) subject 3.

The classification of emotions using micro-expressions is a challenging task due to the issue of multi-scale in muscle movements among the subjects. Some subjects have a broader facial structure and hence, the muscle movements expressed by them affect wider areas, especially around the mouth and eyes. On the other hand, some subjects have a slimmer facial structure or smaller eyes, which will result in different movement patterns, thence creating variable size challenges to the recognition system. To reduce the facial size issue, all input images were resized and cropped to a standard format, in which only a region of interest from each subject will be processed by the system. The standardized facial region is readily available from each of the tested datasets. Yet, the size of the affected muscle movement areas cannot be standardized, especially when multi-datasets of various subject backgrounds are used for validation. Figure 1 shows a few subjects that express a happy emotion, whereby they produce different movement intensity around the mouth regions. In this example, subject 3 produces bigger facial muscle movements compared to subjects 1 and 2. It is also observable that the subjects have different facial structures, which cause different patterns in the muscle excitation, which can be addressed by embedding multi-scale capability in the deep network.

Therefore, multi-scale embedding in the CNN network was recently explored by Sian et al. [6] through simple insertion of the Spatial Pyramid Pooling (SPP) module. However, the authors only tested a basic SPP using two sets of down-pooling kernels using a fixed number of parallel paths. Even more, the multi-scale unit is inserted into the original architecture of VGG-M without modifications or network optimization. In addition to that, they have not explored other multi-scale network configurations apart from the SPP unit. To overcome their suggested work limitations, we analyze and design a comprehensive multi-scale unit addition to a compact network. Two multi-scale approaches are explored that include the SPP and Atrous Spatial Pyramid Pooling (ASPP), which will be optimized in terms of unit placement, number of parallel paths, and down-pooling kernel sets. In addition, we also analyze the network flow of the multi-scale unit through direct and waterfall methodology to produce the best emotion recognition based on micro-expression input cues. Therefore, the following are main contributions of this paper: (i) optimize multi-scale approaches by exploring the optimal position and parallel branches for the SPP and ASPP module, (ii) optimize network flows—either direct or waterfall flows.

The proposed multi-scale networks are discussed in five sections, whereby the following Section 2 provides an overview of various related works, while Section 3 describes the technical details used to classify the emotions using facial micro-expression cues, which also include all the proposed architecture variants. Section 4 discusses the results of the emotion classification experiments, followed by Section 5 which concludes the findings and some plans for future work.

## 2. Recent Works

In general, micro-expression recognition systems are separated into two modules: spotting the maximum instantaneous changes in facial expressions and classifying the emotion behind the spotted micro-expression frame. The spotting approach assumes that a single frame information, which is the apex frame, is enough to detect the right emotion. On the other hand, if a long-video format is used, the spotting module aims to recognize three crucial frames, namely the onset, apex, and offset frames, which are then used to detect the presence of micro-expressions. 

In ref. [7], Davison et al. detected micro-expressions via histogram-oriented gradient by labeling the frames as the true positive detection if the frame sequence is less than 100 frames, which also includes noise issues from video flicker and fixation. Then, a false positive case is identified when the detected motion sequence is not encoded into the respective class. Their simulation results, which were tested on the SAMM database, produced accuracy, Recall Rate, and F1 score of 0.70, 0.84, and 0.76, respectively. According to the research in ref. [8], the combination of optical strain and optical flow magnitudes can further improve the performance of automated emotion recognition using micro-expressions, which have been verified using both SMIC and CASME II datasets. In ref. [2], Liu et al. employed a unique optical flow technique known as MDMO to better extract the textural information of the images. Then, an affine transformation was used to eliminate any subtlety of illumination and head motions. The facial areas were also subdivided into several regions of interest (ROIs), which were then fed to an SVM classifier to detect the genuine emotion class. One of the papers that popularizes the usage of the apex frame as the sole input for emotion classification is the work by Liong et al. [3]. They utilized the Bi-WOOF method to extract the important features in the apex frame. Then, OffApexNet was proposed in ref. [9] as a hybrid approach, whereby this network employs two frames of information to represent the micro-expression: onset and apex frames. Then, the computed optical flow features from these two frames were passed to a CNN model for optimal feature extraction. 

Deep learning has shown promising results in various domains of study in recent years [10,11,12], and it has also been used successfully in micro-expression recognition. Kim et al. [13] proposed a combination between CNN and long short-term memory (LSTM) to capture the spatio-temporal information in a video to locate and recognize micro-expressions. The spatial features of facial expressions were first analyzed using CNN that covers all expressions, which are then passed to the LSTM to extract temporal relationships of the CNN inputs. Khor et al. [14] presented a unique CNN-LSTM model through the Enriched Long-Term Recurrent Convolutional Network (ELRCN). Their approach utilized both optical flow and optical strain characteristics as the inputs to model the minute facial muscle movements. This combination of CNN-LSTM has been proven to be robust in extracting both the optimal temporal and spatial features from the tested videos [15,16,17]. After the features were extracted, the emotion was categorized using a conventional machine learning approach through Support Vector Machines (SVM). For micro-expression recognition, Shaheen et al. [18] proposed a framework for an emotion recognition system that treats emotions as generalized ideas abstracted from sentences by incorporating compositional, syntactic, and semantic analysis. Erenel et al. [19] developed and compared a new feature selection approach for emotion classification to various feature reduction techniques, including chi-square, Gini-text, and delta. The proposed approach, known as the relevance score, was shown to improve emotion classification. 

Peng et al. [20] suggested a dual-template CNN model based on the optical flows extracted from successive micro-expression sequences. However, extracting their multiple optical flow input requires much computation, which significantly reduces the dual-template CNN model’s efficiency. The optical flow data from the entire video need to be retrieved first before they are supplied to the CNN feature extractor. Then, a new automated micro-expression analysis technique, which is called Flownet 2.0 [21], was used by Li et al. [22] to improve a dual-template CNN model performance, yet the performance is still inferior to the conventional approaches [23]. Kumar et al. [24] then employed a method based on frequency domain to delete low-intensity expression frames. In their paper, the frames with the least amount of texture variance are defined as the low-intensity frames. Significant motion will magnify the emotion image that was created from the remaining high-intensity frames. The emotions are then classified by passing through all these high-intensity frames into the respective CNN model. SPP was initially introduced by He et al. [25], and has been effectively applied to various semantic segmentation tasks [26], anti-spoofing applications [27], expression analysis systems [6], and many other automated systems in the computer vision literature. Meanwhile, ASPP was originally proposed by Chen et al. [28], and demonstrated success in a range of works that include object detection [29], image segmentation [30,31], image classification [32], etc.

## 3. Methodology 

Firstly, a compact base CNN model with five convolutional layers was proposed. It is hard to optimize a deeper network because of the limited availability of the database. Then, the optimal design of the multi-scale modules that include exploration of SPP and ASPP modules was implemented by considering various configurations of parallel paths and module placement positions. Lastly, optimal network flow selection for both SPP and ASPP modules between direct and waterfall flows was experimented to produce the best-performing emotion classification system.

### 3.1. Dataset

A crucial prerequisite for developing a micro-expression-based emotion classification system using a deep learning network is the availability of sufficient labeled training data. In general, our primary emotions are classified into six different categories, namely, angry, disgusted, scared, happy, sad, and surprise. However, in this study, only three types of emotion are used, whereby several emotions are combined into either positive, negative, or surprise. In addition to that, a combined dataset from three available online databases was used in this study, namely CASME II, SAMM, and SMIC, which also limits the emotion categories to three classes only. Table 1 shows the number of samples for each dataset used in this study.

The Chinese Academy of Micro-Expression Sciences (CASME II) is one of the latest versions of the CASME family dataset that was developed by Yan et al. [33], which contains 247 micro-expressions from 26 subjects. Each of the 247 micro-expression samples has been annotated into one of the five emotion classes, namely happy, disgusted, shocked, oppressive, and others. All video sequences of the facial micro-expressions were recorded using a high-speed camera with a frame rate of 200 frames per second (fps) with a relatively low resolution of 280 × 340 pixels. The expressions in the CASME II database were labeled based on a combined assessment of the Action Unit (AU), participant reports, and video content. In this study, only 145 samples were used due to the emotion class availability, which is then separated into three emotion categories: positive (happy), negative (repression, disgust), and surprise.

The second dataset, Spontaneous Actions and Micro-Movement (SAMM), was developed by Davison et al. [34], consisting of 156 samples of micro-expressions. The data were collected from 32 subjects with an average age of 33.24 years that come from diverse ethnicities. This dataset originally had seven types of micro-expression-based emotions: hatred, disgust, surprise, fear, sadness, anger, and happiness. Contrary to the CASME II dataset, the SAMM dataset was recorded using a high-speed camera of 200 fps with a resolution of 2040 × 1088 pixels. This dataset also comes with an annotated frame index for the onset, apex, and offset frames. In this study, only 133 micro-expression samples were used, which were then recategorized into three types of emotions: positive (happy), negative (fear, disgust, hatred, sadness, and anger), and surprise.

The last dataset, Spontaneous Micro-Expression Corpus (SMIC), was developed by Li et al. [35], and consists of three imaging subtypes, namely HS-SMIC, VIS-SMIC, and NIR-SMIC. There are a total of 164 micro-expression samples taken from 16 subjects for the HS-SMIC subtype, whereas only 71 samples from 7 subjects were available in the VIS-SMIC and NIR-SMIC datasets. Again, the samples from these three SMIC subsets were divided into three micro-expressions categories, namely positive, negative, and surprise. These micro-expression samples also come with annotated frame index information for the onset and apex frames. 

### 3.2. CNN Architecture Model

This study uses compact CNN architecture to extract sophisticated micro-expression information to classify the emotion categories into three classes. Before a multi-scale module is added, an optimized compact network needs to be finalized so that the multi-scale experiments can be processed effectively. The base model is derived from optimal hierarchical spatial features using multiple building blocks such as convolutional, pooling, and fully connected (FC) layers. The final base model will include five convolutional layers, three pooling layers, and three FC layers. The pooling layer task is to reduce the feature map size generated by the convolutional layer. Then, the FC layer takes all the latent variables and performs dense connections from the previous layer.

Then, an optimal set of hyperparameters needs to be configured due to the compact nature of the model that has a higher likelihood to overfit when the training data availability is limited. If the overfitting problem occurs, the training accuracy will be relatively high, while the test accuracy will return a much poorer performance. In other words, the model learns with limited generalization capability, whereby the distractions that are not needed also fit into the model during the training phase. Table 2 shows the network architecture of the base CNN model used for multi-scale integration experiments.

Based on Table 2, the first and second convolutional layers use kernel depths of 96 and 256, respectively. In comparison, the kernel depth for convolutional operation in the third, fourth, and fifth layers is set to 512. Then, the output size of the fully connected (FC) layers, FC1 and FC2, are set to 128 units, while FC3 only uses three output nodes because of the three emotion classes. All input networks are fed with the optic flow images that are adjusted to a size of 75 × 75 pixels, which is the input requirement for the first convolutional layer (Conv1). The activation function for all convolutional layers, FC1 and FC2, is set to Rectified Linear Unit (ReLu), whereas the FC3 activation layer utilizes the Softmax function to make the final classification. This study generally focuses on modifying the proposed architecture by inserting new layers into it, namely the SPP layer and the ASPP layer.

### 3.3. Emotion Classification Based on the SPP Module 

**Figure 2:**
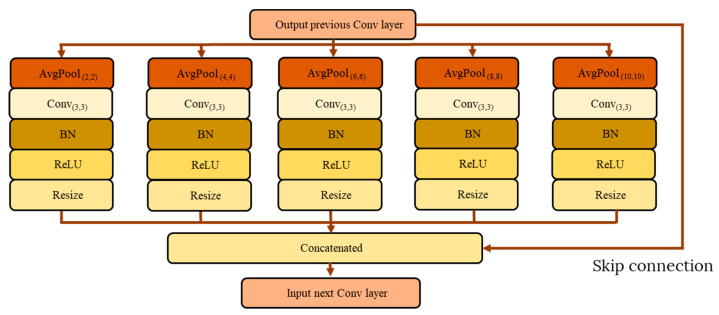
Basic SPP module architecture.

SPP is a multi-scale feature pooling module that uses repeated down-pooling information to create parallel branches to extract features of various sizes. Each of the new branches will focus on a smaller set of features. The SPP modules generally consist of four parallel layers, and each layer has a different feature map size, derived from the same original input source. For each parallel branch, the feature extraction process will consist of a convolution layer, average pooling layer, batch normalization, and ReLU activation function. The kernel size of average pooling to down-sample the feature maps varies between different scales. Then, the output of each parallel branch will undergo a resizing process by scaling them to match the input source size so that all outputs can be concatenated together. A skip connection layer is also added to bring forward the original feature map to further enrich the multi-scale feature extraction module. Figure 2 shows the general architecture of an SPP module with four parallel branches. 

**Figure 3:**
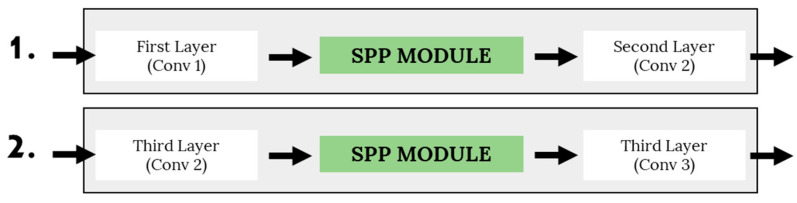
Two placement strategies of the SPP module in the base CNN model.

Several variants of the SPP module have been developed to produce an optimal compact network to recognize human emotion through micro-expression input. In this study, the number of optimal parallel branches in the SPP modules is experimented with, coupled with the optimal placement of the module. Overall, there are eight variants of SPP module architectures being developed and tested. The differences between these variants are in terms of the optimal number of parallel path modules, the kernel size of average pooling, and the position of the module placement. Specifically, this study defines the average pooling parameters as (2, 4, 6, 8, 10) pool size, which produces pool sizes of 2 × 2, 4 × 4, 6 × 6, 8 × 8, and 10 × 10, respectively. Then, the fixed-dimensional vectors will be the input for the following convolution layer. Table 3 shows the list of SPP module architectures that were proposed to recognize the emotions, and Figure 3 shows the position of the embedded SPP module on the proposed base CNN model.

### 3.4. Emotion Classification Based on the ASPP Module 

**Figure 4:**
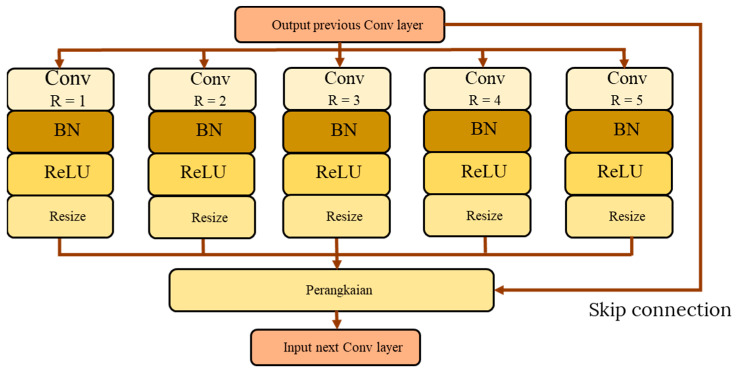
Basic ASPP module architecture.

The original version of the ASPP module was first introduced in [36], which extracts multi-scale features through parallel atrous convolution with different dilation rates. Atrous convolution as applied in the ASPP module allows the convolutional filter to capture larger feature maps by allowing spaces between the filter kernel. The larger the spacing, the bigger the dilation rate, which still retains the same sized convolutional kernel. This sparse concept of filter captures the multi-scale features through varying the atrous rate. For each parallel branch in ASPP, the feature maps will be processed through a sequence of atrous convolutional layer, batch normalization, and ReLU activation function. Atrous convolution resembles the standard convolution operation, except that its kernel will sparsely be expended by adding zero rows and columns weights. Thus, in each dimension of the atrous convolutional filter, a gap of *r − 1* is formed between two consecutive filter values, whereby *r* is the dilation rate. Figure 4 shows a basic ASPP module architecture with five parallel branches. The atrous convolution is defined by Equation (1), where *W[n]* is the output of the atrous convolution, *n* is the index, *k* is the location of the index in the kernel, *r* is the dilation rate that determines the kernel size of the atrous convolution layer, and *f* is the filter weight. Changing the dilation rate can adjust the output size of the resultant feature maps, which will capture different regions of the micro-expressions.

(1) $$W\left[n\right]=\overset{d}{\underset{d=1}{\sum }}x\left[n+r\cdot k\right]f\left[d\right]$$

**Figure 5:**
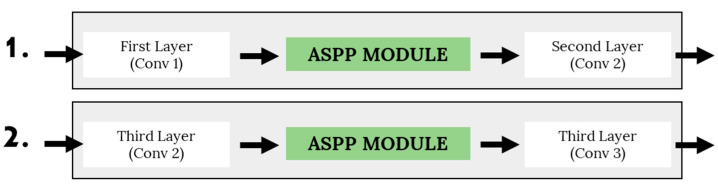
Two placement strategies of the ASPP module in the base CNN model.

There are several variants of the ASPP modules that were developed in this study. In general, and the difference between the variants can be summarized according to the following criteria: (1) the number of parallel pathway modules, (2) the dilation rate used in the atrous convolution layer, and (3) the placement of the ASPP module in the base CNN architecture. Figure 5 shows the possible placement configurations of the ASPP module in the base CNN architecture, while Table 4 shows the summary of network characteristics of the ASPP variants.

### 3.5. Direct and Waterfall for SPP and ASPP Module

**Figure 6:**
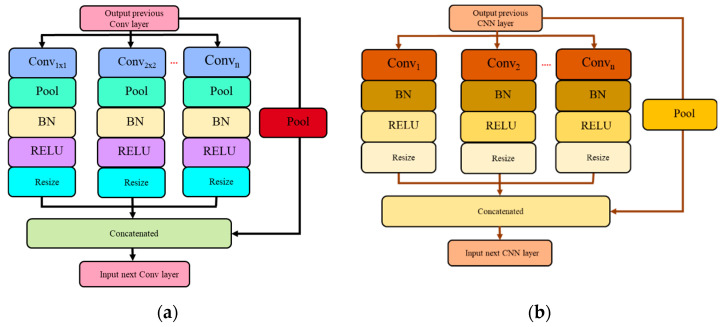
Direct network flow of the SPP and ASPP modules: (**a**) DSPP-Net architecture; (**b**) DASPP-Net architecture.

To further optimize the design of both the SPP and ASPP modules, two types of network flow were experimented with, which are direct and waterfall flows. As a result, this paper introduces a set of four new architecture variants, namely Direct Spatial Pyramid Pooling (DSPP-Net), Waterfall Spatial Pyramid Pooling (WSPP-Net), Direct Atrous Spatial Pyramid Pooling (DASPP-Net), and Waterfall Atrous Spatial Pyramid Pooling (WASPP-Net). A new branch in the direct network flow derives its input feature map from the original input source, while a new branch in the waterfall network flow derives its input from the previous parallel branch that mimics the waterfall flow. The network flow of the direct scheme for both SPP and ASPP architectures are shown in Figure 6. 

**Figure 7:**
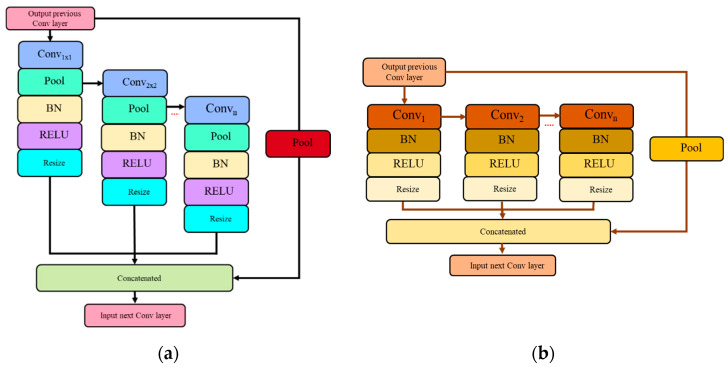
Waterfall network flow for SPP and ASPP modules: (**a**) WSPP-Net architecture; (**b**) WASPP-Net architecture.

The proposed WSPP-Net and WASPP-Net architectures modify the input source of the DSPP-Net and DASPP-Net when a new parallel branch is created. For a set of four parallel branches, DSPP-Net divides the input into four paths through average pooling operators with a set of kernel sizes of 4 × 4, 6 × 6, 8 × 8, and 10 × 10 kernels, coupled with a skip connection of the original input source. On the other hand, for a set of four parallel branches of DASPP-Net, the networks will create four network paths by using a set of atrous convolution operators with different dilation rates of 2, 3, 4, and 5. However, for the WSPP-Net and WASPP-Net, the input source for the next parallel branch will be derived from the previous branch, which acts like a waterfall flow, from which it gets its name, as shown in Figure 7. The waterfall network flow tries to diversify the input source so that the features are extracted by utilizing broader fields-of-view (FOV) while maintaining the same number of parallel branches.

## 4. Results and Discussions

### 4.1. Training Setup

The platform used to execute the experiments was based on an Intel Core i7-4770 coupled with an NVIDIA Titan V video card. In addition to that, the NVIDIA CUDA 10.1 framework and cuDNN 8.0.3 library were used to enable the parallel computation to speed up the training process. The Leave-One-Subject-Out (LOSO) approach was applied in this investigation so that bias among the subjects can be reduced. It is recommended that one subject from all micro-expression datasets should be set aside for testing, and the remaining subjects should be utilized for the training purpose. The performance of all proposed variants will be measured using accuracy and F1 score evaluation metrics. The equation and explanation of the performance metrics are as follows:

1. Accuracy (*Ac*): the ratio of correctly predicted results compared to the number of samples. The formula for calculating the accuracy is shown in Equation (2), where *T_(+ve__)_* is the true positive, *T_(−ve__)_* is the true negative, and *Ts* is the total number of samples.
  (2) $$Ac=\frac{T_{\left(+ve\right)}+T_{\left(-ve\right)}\;}{Ts}\;$$
2. *F*1 *score*: the mean harmonic for recall, *Re*, and precision, *Pr*. It captures a balanced metric between recall and precision metrics with an output range between 0 and 1. If the model has a perfect recall and accuracy values, then its *F*1 *score* is 1, whereas, if one or both recall and accuracy are 0, then its *F*1 *score* will be 0. The *F*1 *score* formulas are shown in Equations (3)–(5), where *F*_(*+ve*)_ indicates the false positive detection, and *F*_(*−ve*)_ indicates the false negative detection.
  (3) $$F1\;score=2\times \frac{Pr\times Re}{Pr+Re}\;$$
  (4) $$Pr=\frac{T_{\left(+ve\right)}}{T_{\left(+ve\right)}+F_{\left(+ve\right)}}\;$$
  (5) $$Re=\frac{T_{\left(+ve\right)}}{T_{\left(+ve\right)}+F_{\left(-ve\right)}}\;,$$

Optimal selection of hyperparameters is essential to control the algorithm during the training process, which will significantly impact the performance of the tested CNN model. Hence, Table 5 shows a list of experimental hyperparameters and their role that include optimizer, learning rate, group size, and number of training samples.

### 4.2. SPP Module Results Based on the Position and Number of Parallel Branches

This section analyses and discusses the outcomes of the suggested SPP module for classifying emotions based on micro-expression cues. Table 6 lists the classification accuracy for the base CNN coupled with various variants of the SPP module that differ in the number of parallel branches used and module placement strategy. The accuracy results of the modified networks tested on the three databases improved significantly with the addition of the SPP module. For SAMM and combined datasets, the best performance is obtained by model VIII, which consists of five parallel branches with a maximum kernel size of 10 × 10 pixels, which are placed after Conv2. The accuracy performance for the SAMM database is 73.23%, while the accuracy performance for the combined datasets is 79.59%. Meanwhile, if only the CASME II dataset is considered, the best accuracy of 91.26% is obtained through models I and VII. The former model uses two parallel branches, placed after Conv1, while the latter model uses four parallel branches, placed after Conv2. Lastly, the accuracy performance tested on the SMIC dataset did not show any improvement when the SPP module was embedded into the base CNN model. 

Table 7 shows the overall classification F1 score findings for the CASME II, SAMM, SMIC, and combined datasets utilizing the suggested SPP variants that focus on the number of parallel paths and module placement strategy. With regards to model VIII, it produced better F1 scores of 0.6939 and 0.5985 tested on the SAMM and the combined datasets, respectively, which are much higher performance values compared to the original base CNN model (0.6621 and 0.5152). While F1 score performance for the CASME II dataset is inconclusive with 0.869, the score performance dropped for all variants when they were tested on the SMIC dataset.

### 4.3. ASPP Module Results Based on the Position and Number of Parallel Branches

The experimental results of the emotion classification for various configurations of the parallel paths and placement strategy of the ASPP module are shown in Table 8. As the findings demonstrate, the base CNN model embedded with the ASPP module consistently outperforms the original base CNN model for all validation datasets, except for the SMIC dataset. Model V improves the accuracy of emotion classification with consistent gains of 6.06% and 1.44%, tested on SAMM and combined datasets, respectively. Then, the increasing pattern of the result can also be observed for the CASME II dataset using models VI and VII. However, the models perform poorly for the SMIC dataset, as they do not produce performance increments in any embedded ASPP model like the other two datasets.

Table 9 illustrates the F1 score performances for emotion classification based on the number of parallel paths and placement strategy of the ASPP module. For model V, its F1 score surpasses the original base CNN model by 0.025 tested on the combined dataset and 0.0909 tested on the SAMM dataset. The performance using the CASME II dataset also improved by 0.0348 when models VI and VII were used, but all variants performed poorly when tested on the SMIC dataset alone.

### 4.4. SPP and ASPP Module Using Direct and Waterfall Network Flows

**Figure 8:**
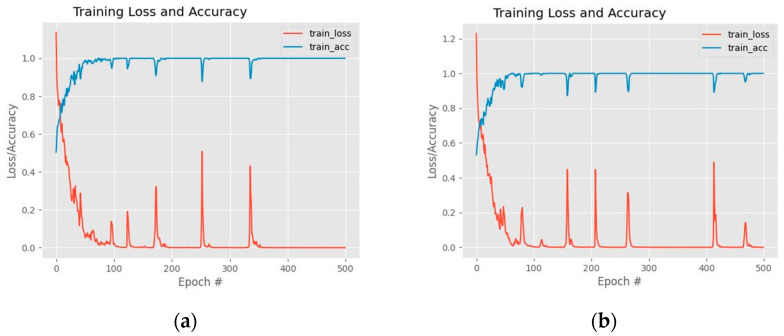
The training graph performance: (**a**) DSPP-Net architecture; (**b**) WSPP-Net architecture.

Table 10 compares the emotion classification accuracy of DSPP-Net and WSPP-Net architectures. The WSPP-Net performance is noticeably higher compared to DSPP-Net. The highest accuracy is obtained by WASPP-Net when it is tested using CASME II, SAMM, SMIC, and the combined dataset with performance values of 92.18%, 72.73%, 75.61%, and 80.2%, respectively. In contrast, the accuracy performance of the WSPP-Net remains the same as the original base model and hence, the embedded multi-scale unit does not contribute to the betterment of the network performance. On the other hand, the accuracy performance of DSSP-Net is slightly lower, by 1.22%, when it is tested on the SMIC dataset. Figure 8 illustrates the training graph performance between DSPP-Net and WSPP-Net architectures. 

**Figure 9:**
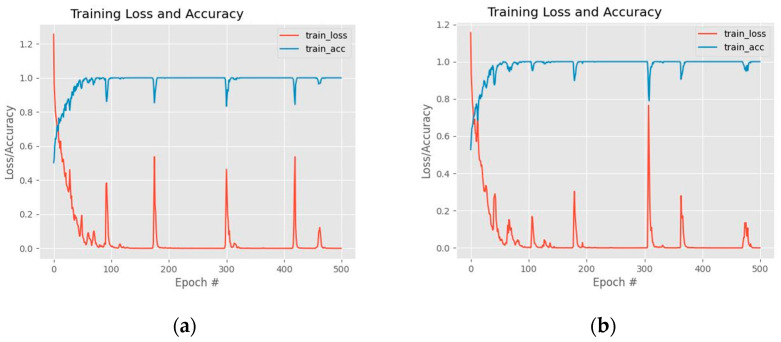
The training graph performance: (**a**) DASPP-Net architecture; (**b**) WASPP-Net architecture.

The overall performance results of the DASPP-Net and WASPP-Net are illustrated in Table 11. According to these findings, the accuracy of emotion classification using micro-expression cues has increased compared to the base CNN model through WASPP-Net and DASPP-Net, for all dataset categories. In general, WASPP-Net returns a better emotion classification performance compared to the DASPP-Net. Specifically, if CASME II, SAMM, SMIC, and combined datasets are considered, the WASPP-Net achieves performance increments of 3.67%, 3.58%, 2.03%, and 3.02%, respectively, when it is compared to the base CNN model. After considering all experiments, it is noticeable that both SPP and ASPP modules will produce promising results when the waterfall network flow is used, instead of the direct flow. Figure 9 shows the training graph performance between DASPP-Net and WASPP-Net architectures. Furthermore, Table 12 illustrates the timing comparison between the execution time of DASPP-Net and WASPP-Net architectures. 

Apart from the classification performance, we also analyzed the timing performance for each proposed method. Table 12 compares the execution time between DSPP-Net, WSPP-Net, DASPP-Net, and WASPP-Net architectures. From this result, the execution time for WSPP-Net architecture is the fastest compared to other architecture, which can be processed with up to 591 frames per second (fps) during the training phase. We used the “Time” library to measure the execution time, which is processed using a high-end GPU of Nvidia Titan X. In general, the waterfall flow configuration for both SPP and ASPP architectures is the faster version compared to the direct flow configuration. The execution time for WASPP-Net is faster than DASPP-Net, whereby the training time for WASPP-Net consumes only 447 s compared to DASPP-Net, which consumes 548 s. The slowest architecture among them is DASPP-Net, which can process a relatively lower timing performance of just 400 fps. 

### 4.5. Benchmark to the State-of-the-Art the Algorithms

Table 13 shows the performance comparison between the proposed method, WASPP-Net, and the state-of-the-art CNN models. The results show that WASPP-Net produced the highest accuracy of 80.50% and an F1 score of 0.7075 compared to the other state-of-the-art CNN methods. The second-best method is OffApexNet, in which WASPP-Net shares a lot of base architecture similarities. Hence, we can also deduce that the performance increment is due to the addition of an optimized multi-scale unit, which improves the accuracy from 78.38% to 80.50%. However, a simple application of VGG-M without any network modification will produce a low accuracy, as the size of the feature maps is too small for the latter layers, which results in low accuracy and an F1 score of 72.34% and 0.5850, respectively. It is observable that the third, fourth, and fifth layers do not really carry much information, as the feature maps are at most 3 × 3 pixels.

In addition to that, it is interesting to note that the increment in the number of parameters or network size does not always produce a better emotion recognition performance. This is proven in the previous subsections, whereby more parallel paths will not produce a better performance. In fact, for WASPP-Net, three parallel branches are a better configuration compared to the five parallel branches. Table 14 lists the total number of network parameters for each proposed architecture model. Even though DSPP-Net has higher parameters of 8,378,659 compared to WASPP-Net with 8,117,794 parameters, WASPP-Net still produced the best emotion classification among them.

## 5. Conclusions

This work proposed multiple variants of multi-scale deep learning models for emotion classification using micro-expression cues. Two main network strategies either using Spatial Pyramid Pooling or Atrous Spatial Pyramid Pooling (ASPP) were shown, where both of them are optimized according to the number of parallel branches as well as the module placement scheme. The proposed methodologies were evaluated using three publicly available spontaneous micro-expression databases (CASME II, SMIC, and SAMM), which are readily downloadable online. As demonstrated in the experiments, the suggested techniques have the potential to greatly improve the accuracy of micro-expression-based emotion classification. However, it is also noticeable that the multi-scale module does not improve the classification performance when it is tested on the SMIC dataset. The main reason for this abnormality is due to the absence of an annotated apex frame index by the dataset provider, which makes it impossible to provide representative information on the subject matter. Furthermore, this paper also concludes that the WASPP-Net that utilizes waterflow network flow with the ASPP module produced the best classification performance compared to the original base CNN model, with an overall accuracy of 80.5%. For future works, attention-based mechanisms and feedforward paths can be embedded into the base CNN model to increase network capability in locating the regions of interest as well as improving the carry-over information from the early layers.

## Figures and Tables

**Table 1:** The number of samples for the tested datasets.

Types of Emotion	Combined	CASME II	SAMM	SMIC
Positive	109	32	26	51
Negative	250	88	92	70
Surprise	83	25	14	43
Total	441	145	132	164

**Table 2:** Network architecture of the proposed base CNN model.

Layer	Size of Kernel	Stride	Padding	Size of Output	Activation Function
Conv1	7 × 7	1	1	96 × 69 × 69	ReLu
Conv2	5 × 5	1	1	256 × 65 × 65	ReLu
Conv3	3 × 3	1	0	512 × 65 × 65	ReLu
Pool3	3 × 3	2	1	512 × 32 × 32	-
Conv4	3 × 3	1	0	512 × 32 × 32	ReLu
Pool4	3 × 3	2	1	512 × 16 × 16	-
Conv5	3 × 3	1	0	512 × 16 × 16	ReLu
Pool5	3 × 3	2	1	512 × 8 × 8	-
FC1	-	-	-	128	ReLu
FC2	-	-	-	128	ReLu
FC3	-	-	-	3	Softmax

**Table 3:** List of the SPP module architecture variants.

SPP Model	Number of Parallel Paths	Maximum Kernel Size	Position
I	2 SPP	4 × 4	After Conv1
II	3 SPP	6 × 6	After Conv1
III	4 SPP	8 × 8	After Conv1
IV	5 SPP	10 × 10	After Conv1
V	2 SPP	4 × 4	After Conv2
VI	3 SPP	6 × 6	After Conv2
VII	4 SPP	8 × 8	After Conv2
VIII	5 SPP	10 × 10	After Conv2

**Table 4:** List of ASPP module architecture.

ASPP Model	Number of Parallel Paths	Maximum Dilation Rate	Position
I	2 ASPP	2	After Conv1
II	3 ASPP	3	After Conv1
III	4 ASPP	4	After Conv1
IV	5 ASPP	5	After Conv1
V	2 ASPP	2	After Conv2
VI	3 ASPP	3	After Conv2
VII	4 ASPP	4	After Conv2
VIII	5 ASPP	5	After Conv2

**Table 5:** List of experimental hyperparameters.

Hyperparameter	Type/Value	Function
Optimizer	Adam [37]	Update parameters such as weights and learning rates to reduce losses
Learning rate	0.0001	Update weights
Batch size	32	Number of samples taken to update model parameters
Number of training samples	Leave-One-Subject-Out (LOSO)	The combined number of samples used

**Table 6:** Emotion classification accuracy evaluated based on number of parallel paths and placement position of the SPP module.

Types of Datasets		Accuracy (%)							
	Original (Without SPP Module)	Types of SPP Model							
		I	II	III	IV	V	VI	VII	VIII
Combined	77.48	77.63	77.32	77.63	77.48	77.93	78.23	77.48	79.59
CASME II	88.51	91.26	87.59	88.51	88.51	89.43	87.13	91.26	89.89
SAMM	67.68	67.17	69.7	71.21	70.2	69.7	72.73	67.68	73.23
SMIC	75.61	73.98	74.39	73.17	73.58	74.39	74.8	73.17	75.61

**Table 7:** Overall emotion classification F1 score results evaluated based on number of parallel paths and placement position of the SPP module.

Types of Datasets		F1 Score							
	Original (Without SPP Module)	Types of SPP Model							
		I	II	III	IV	V	VI	VII	VIII
Combined	0.6621	0.6644	0.6599	0.6644	0.6621	0.6689	0.6735	0.6621	0.6939
CASME II	0.8276	0.869	0.8138	0.8276	0.8276	0.8414	0.8069	0.869	0.8483
SAMM	0.5152	0.5076	0.5455	0.5682	0.553	0.5455	0.5909	0.5152	0.5985
SMIC	0.6441	0.6098	0.6159	0.5976	0.6037	0.6159	0.622	0.5976	0.6341

**Table 8:** Emotion classification accuracy evaluated based on number of parallel paths and placement position of ASPP module.

Types of Datasets		Accuracy (%)							
	Original (Without ASPP Module)	Types of ASPP Model							
		I	II	III	IV	V	VI	VII	VIII
Combined	77.48	76.11	77.02	76.11	78.53	79.14	78.08	77.48	77.63
CASME II	88.51	89.89	89.97	86.67	89.42	88.97	90.8	90.8	87.59
SAMM	67.68	66.16	70.71	70.71	71.21	73.74	69.19	70.2	70.2
SMIC	75.61	71.95	71.54	71.14	74.8	74.8	73.98	71.54	74.8

**Table 9:** Overall precision of emotion classification F1 score results evaluated based on number of parallel paths and placement position of the ASPP module.

Types of Datasets		F1 Score							
	Original (Without ASPP Module)	Types of ASPP Model							
		I	II	III	IV	V	VI	VII	VIII
Combined	0.6621	0.6417	0.6553	0.6417	0.678	0.6871	0.6712	0.6621	0.6644
CASME II	0.8276	0.8483	0.8345	0.80	0.8414	0.8345	0.8621	0.8621	0.8138
SAMM	0.5152	0.4924	0.5606	0.5606	0.5682	0.6061	0.5379	0.553	0.553
SMIC	0.6441	0.5793	0.5732	0.5671	0.622	0.622	0.6098	0.5732	0.622

**Table 10:** Comparison of emotion classification accuracy on DSPP-Net and WSPP-Net architectures.

Types of Datasets	Accuracy (%)		
	Original (Without SPP Module)	DSPP-Net	WSPP-Net
Combined	77.48	77.93	80.20
CASME II	88.51	89.43	92.18
SAMM	67.68	69.7	72.73
SMIC	75.61	74.39	75.61

**Table 11:** Comparison of emotion classification accuracy on DASPP-Net and WASPP-Net architectures.

Types of Datasets	Accuracy (%)		
	Original (Without ASPP Module)	DASPP-Net	WASPP-Net
Combined	77.48	78.08	80.50
CASME II	88.51	90.8	92.18
SAMM	67.68	69.19	71.21
SMIC	75.61	73.98	77.64

**Table 12:** Execution time comparison between DSPP-Net, WSPP-Net, DASPP-Net, and WASPP-Net architectures.

Type of Architecture	Training Time Per Subject (s)	Execution Time (Frames Per Second)
Original (Without SPP/ASPP Module)	520	418
DSPP-Net	431	510
WSPP-Net	370	591
DASPP-Net	548	400
WASPP-Net	447	460

**Table 13:** Performance comparison to the state-of-the-art CNN models.

Method	Accuracy (%)	F1-Score
VGG-M	72.34	0.5850
DualInception	73.09	0.5964
AlexNet	75.51	0.6327
STSTNet	77.48	0.6621
OffApexNet	78.38	0.6757
WASPP-Net	80.50	0.7075

**Table 14:** Number of network parameter for each architecture model.

Types of Models	Number of Parameter
DSPP-Net	8,378,659
WSPP-Net	8,231,203
DASPP-Net	8,378,659
WASPP-Net	8,117,794

## Data Availability

The dataset can be downloaded from http://fu.psych.ac.cn/CASME/casme2-en.php (accessed on 20 April 2022).
